# Integrating Whole-Genome Sequencing in Clinical Genetics: A Novel Disruptive Structural Rearrangement Identified in the Dystrophin Gene (*DMD*)

**DOI:** 10.3390/ijms23010059

**Published:** 2021-12-22

**Authors:** Ana Gonçalves, Ana Fortuna, Yavuz Ariyurek, Márcia E. Oliveira, Goreti Nadais, Jorge Pinheiro, Johan T. den Dunnen, Mário Sousa, Jorge Oliveira, Rosário Santos

**Affiliations:** 1 Unidade de Genética Molecular, Centro de Genética Médica Doutor Jacinto Magalhães, Centro Hospitalar Universitário do Porto (CHUPorto), 4099-028 Porto, Portugal; ana.goncalves@chporto.min.saude.pt (A.G.); marcia.oliveira@chporto.min-saude.pt (M.E.O.); jorgemsmoliveira@gmail.com (J.O.); 2 Unidade Multidisciplinar de Investigação Biomédica (UMIB), Instituto de Ciências Biomédicas Abel Salazar (ICBAS) e Laboratório Para a Investigação Integrativa e Translacional em Saúde Populacional (ITR), Universidade do Porto, 4050-313 Porto, Portugal; ana.fortuna@chporto.min-saude.pt (A.F.); msousa@icbas.up.pt (M.S.); 3 Unidade de Genética Médica, Centro de Genética Médica Douto Jacinto Magalhães, Centro Hospitalar Universitário do Porto (CHUPorto), 4099-028 Porto, Portugal; 4 Leiden Genome Technology Center, Leiden University Medical Center, 2333 ZA Leiden, The Netherlands; Y.Ariyurek@lumc.nl (Y.A.); ddunnen@humgen.nl (J.T.d.D.); 5 Serviço de Neurologia, Centro Hospitalar de São João, 4200-319 Porto, Portugal; g.nadais@hotmail.com; 6 Serviço de Anatomia Patológica, Centro Hospitalar de São João, 4200-319 Porto, Portugal; jorge.nature@gmail.com; 7 Departments of Human Genetics and Clinical Genetics, Leiden University Medical Center, 2333 ZA Leiden, The Netherlands; 8 Departamento de Microscopia, Laboratório de Biologia Celular, Instituto de Ciências Biomédicas Abel Salazar (ICBAS), Universidade do Porto, 4050-313 Porto, Portugal

**Keywords:** DMD, whole genome sequencing (WGS), inversion, dystrophinopathies

## Abstract

While in most patients the identification of genetic alterations causing dystrophinopathies is a relatively straightforward task, a significant number require genomic and transcriptomic approaches that go beyond a routine diagnostic set-up. In this work, we present a Becker Muscular Dystrophy patient with elevated creatinine kinase levels, progressive muscle weakness, mild intellectual disability and a muscle biopsy showing dystrophic features and irregular dystrophin labelling. Routine molecular techniques (Southern-blot analysis, multiplex PCR, MLPA and genomic DNA sequencing) failed to detect a defect in the *DMD* gene. Muscle *DMD* transcript analysis (RT-PCR and cDNA-MLPA) showed the absence of exons 75 to 79, seen to be present at the genomic level. These results prompted the application of low-coverage linked-read whole-genome sequencing (WGS), revealing a possible rearrangement involving *DMD* intron 74 and a region located upstream of the *PRDX4* gene. Breakpoint PCR and Sanger sequencing confirmed the presence of a ~8 Mb genomic inversion. Aberrant *DMD* transcripts were subsequently identified, some of which contained segments from the region upstream of *PRDX4*. Besides expanding the mutational spectrum of the disorder, this study reinforces the importance of transcript analysis in the diagnosis of dystrophinopathies and shows how WGS has a legitimate role in clinical laboratory genetics.

## 1. Introduction

Duchenne and Becker muscular dystrophies (D/BMD) are allelic disorders caused by pathogenic variants in the *DMD* gene (Xp21.2-p21.1). This gene, the largest known in the human genome, comprises a total of 79 exons and extremely large introns, in all spanning a genomic segment of around 2500 kb. Given these unique characteristics, the *DMD* gene is particularly prone to genomic rearrangements, mainly intragenic multi-exonic deletions or duplications (~80% of D/BMD cases) [1]; *DMD* gene variant database at www.LOVD.nl/DMD (accessed on 25 September 2021). In the remaining cases, single nucleotide variants are often found, whereas more complex genomic rearrangements, such as intra-chromosomal inversions or translocations coincident with the *DMD* gene, are much rarer mutational events (www.LOVD.nl/DMD). These disease-causing variants lead to a deficiency of dystrophin—a cytoskeletal protein expressed mainly in skeletal muscle and heart, and to a lesser extent in the brain and retina (reviewed in [2]). The deficiency of this protein ultimately translates into progressive muscle weakness, delay and eventually regression of motor development milestones, respiratory insufficiency and cardiomyopathy. In addition to the prominent muscle pathology, D/BMD is also characterized by cognitive and behavioral problems, as nearly 30% of DMD patients show cognitive impairment and 40% have reading difficulties [3].

In recent years, new therapeutic strategies have been developed to modulate the phenotype of DMD patients. While some of these therapies aim to improve muscle function by targeting pathways involved in DMD pathogenesis (anti-inflammatory, antioxidant and anti-fibrosis compounds, for instance), others focus on restoring dystrophin expression, with approaches such as stop codon read-through, exon skipping (with antisense oligonucleotides), gene-addition (vector-mediated microdystrophin), myoblast transplantation and genome editing (CRISPR-Cas9 mediated) [4]. Yet, in many cases, the adequacy and effectiveness of each strategy demands the detailed molecular characterization of the underlying genetic defect in the patients.

We previously reported the detailed genetic characterization of 308 dystrophinopathy patients in 284 unrelated families, where 175 distinct *DMD* pathogenic variants were identified [5]. A complex mutational event involving the deep-intronic insertion of a full-length LINE-1 element in the *DMD* gene was also recently reported by our group, adding to the diversity of genetic singularities that can be observed in this gene [6]. To date, from a cohort of almost 600 referrals with clinical suspicion of D/BMD, over 100 unrelated patients remain unsolved at the genetic level.

The detailed genetic characterization of a novel complex rearrangement identified in one such case is presented in this report. The approach included transcript analysis as well as breakpoint mapping resorting to low coverage whole-genome sequencing (WGS).

## 2. Results

We present the genetic characterization of a BMD patient with a long-standing clinical follow-up and diagnostic workup that spanned almost two decades. The patient’s clinical features and the immunohistochemical (IHC) studies performed on the muscle biopsy were compatible with BMD, encouraging continued genetic investigation targeting the *DMD* locus (Supplementary Material SI).

Initial genomic studies, resorting to Multiplex Ligation-Probe Amplification (MLPA) and sequencing of *DMD* coding regions, excluded the most common disease-causing variants: large deletions or duplications, and changes detectable at the nucleotide level (Supplementary Material SII). Multiplex western-blot analysis performed in the patient’s muscle tissue reinforced the involvement of dystrophin, showing the absence of labelling for two of the targeted dystrophin isotopes (Supplementary Material SIII). There was also a lack of labelling for alpha- and beta-sarcoglycans, which were considered a secondary deficiency consequent to the absence of dystrophin.

In order to sequence the *DMD* transcripts, cDNA obtained from the patient’s second biopsy was amplified as several overlapping fragments covering the entire *DMD* coding sequence. However, one of the expected amplicons, corresponding to exons 67 to 79, consistently failed to amplify. To clarify this absence of PCR amplification, and considering that no deletion was observed at the gDNA level, MLPA was performed on the patients’ muscle cDNA sample. Results revealed the complete absence of signal for the probes that recognize exons 75, 76, 77 and 79 (Supplementary Material SII).

The most likely explanation for these combined results was the presence of a complex rearrangement involving the terminal region of the *DMD* gene.

**Figure 1:**
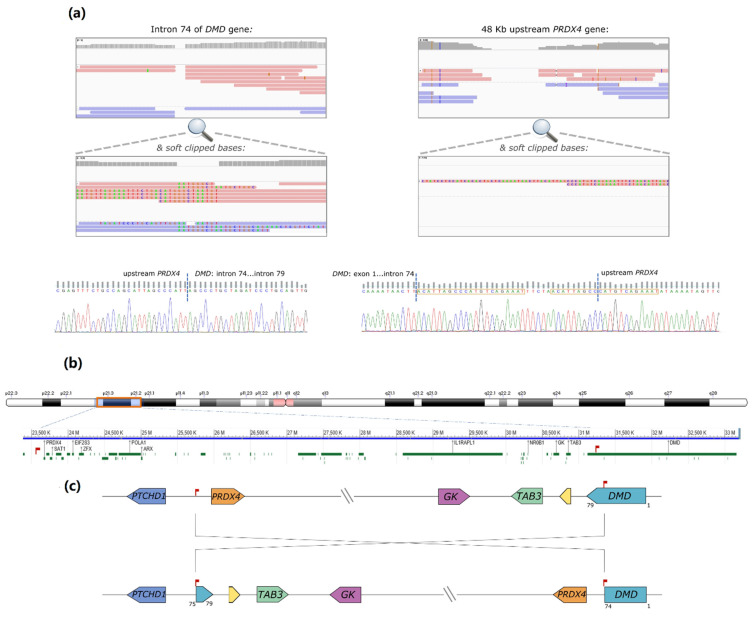
(**a**) Low coverage WGS results suggesting a genomic inversion. Visual BAM file inspection showed a possible breakpoint within intron 74 of *DMD* (1A). Some reads contained soft-clipped bases with homology with a region 48 kb upstream of the *PRDX4* gene (Xp22.1). Correspondingly, some partially aligned reads in the *PRDX4* region showed homology with inverted intron 74 *DMD* sequences; (**b**) Inversion breakpoints were confirmed by Sanger sequencing. In breakpoint 2 (right) the inversion originated a 21 bp duplication (boxed in orange) and the inclusion of a short sequence with no homology to the reference genome. There was also a loss of 9 bp in *DMD* intron 74 and of 3 bp in the upstream *PRDX4* region; (**c**) Schematic representation of the inversion at the genomic level. The inversion involves Xp22.1 to Xp21.2 (~8 Mb), comprising 74 genes. Breakpoints are localized 48 kb upstream of the *PRDX4* gene (breakpoint 1) and within intron 74 of the *DMD* gene (breakpoint 2).

Considering the size and complexity of *DMD*, the strategy used for the breakpoint mapping, towards the identification of a potential mutational event of this nature, consisted in performing low-coverage WGS. The automated calling of structural variants (SV) from the WGS data was inconclusive, as no rearrangements were detected involving *DMD*. However, upon visual scrutiny of the binary alignment map (BAM) file using the Integrative Genomics Viewer (IGV) tool (Broad Institute, [7]), a possible breakpoint was identified within *DMD* intron 74 (chrX:31186159-31186150, Figure 1a). Some reads contained soft-clipped bases with homology to an intergenic region lying 48 kb upstream of the *PRDX4* gene (chrX: 23,635,316-23,635,381 in Xp22.1). Correspondingly, some partially aligned reads in the *PRDX4* region showed homology to inverted *DMD* intron 74 sequences (Figure 1b). Both breakpoints were subsequently confirmed by Sanger sequencing. In one of the breakpoints, the inversion originated a 21 base pairs (bp) duplication and the inclusion of a short sequence (TTCTA) with no homology to the reference genome (Figure 1c). Noteworthy was the loss of only 10 bp in *DMD* intron 74 and 4 bp in the upstream region of *PRDX4*. According to HGVS nomenclature, the variant is described as NC_000023.11:g. [23617262_23617265del;23617266_31168033inv;31168034_31168042delinsACATTAGCCCATGTCAGAAATTTCTAACATTAGCC].

**Figure 2:**
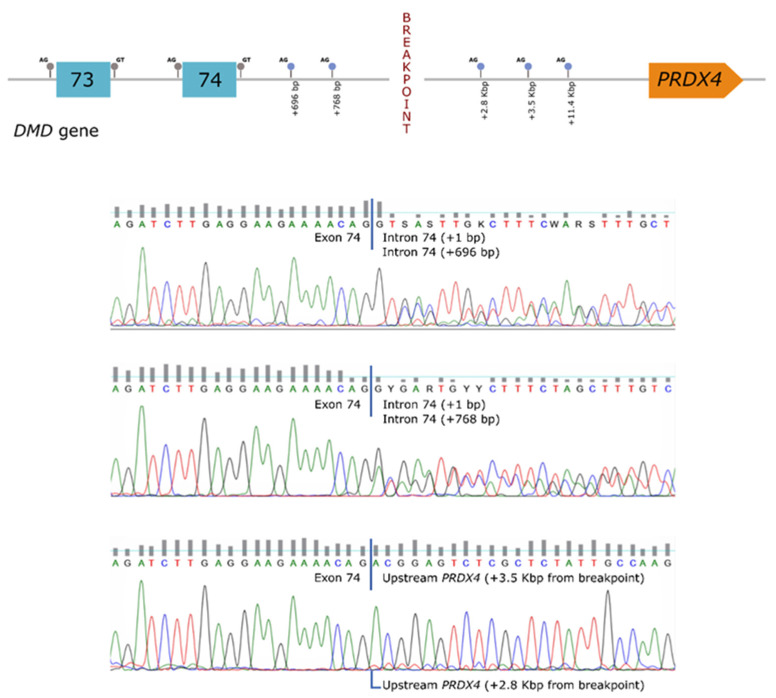
Transcript analysis. Several transcripts were detected in the patient, representing the use of cryptic splice-sites located both in the *DMD* gene (intron 74), as well as in the region upstream *PRDX4*.

In order to establish a correlation between the genomic rearrangement and the dystrophin deficiency observed in the patient, further studies were carried out at the mRNA level. These analyses revealed the presence of several aberrant transcripts, corresponding to the use of cryptic splice-sites located both in the *DMD* gene (intron 74) as well as in the region upstream of *PRDX4* (hybrid transcripts) (Figure 2).

Inversion-specific PCR showed that this structural variant was inherited from the patient’s mother (Supplementary Material SIV). The inversion was not detected in a cohort of 80 other patients suspected to have D/BMD.

## 3. Discussion

A comprehensive analytical approach at the genomic and transcript levels enabled the identification of the underlying genetic defect responsible for the patient’s BMD phenotype. The mutational event was an inversion of approximately 8 Mb (from Xp22.1 to Xp21.2) encompassing a total of 74 genes. *DMD* was apparently the only defective gene, with the positional effect of the inversion originating the presence of several aberrant transcripts and the concomitant loss of the C-terminal region of dystrophin.

The patient’s relatively mild muscular phenotype represents an exception to the reading-frame rule [8], which postulates that truncating variants correlate with a severe disease outcome. Indeed, several BMD patients with truncating mutations in the dystrophin C-terminal domain have been described in the literature, suggesting that this region of the protein is less vital for at least some of the functional roles of the muscle isoform [9]. On the other hand, the patient presents mild intellectual disability with some autistic features. Truncating mutations in the C-terminal region affects all dystrophin isoforms, including the shortest brain isoforms, Dp140 and Dp71, proven to be involved with cognitive impairment in a significant number of patients with D/BMD [10,11].

Thorough bioinformatic analysis of the genomic reference sequence data did not reveal significant similarities between the sequences, in and around the breakpoint sites, neither were there repetitive or retrotransposable elements that could suggest homologous recombination as the driving force for this rearrangement. Only a short homologous stretch of 5 bp (CCATG) was observed (Supplementary Material SV). This finding is compatible with an event that probably follows a microhomology-mediated (MM) replicative model, such as fork stalling and template switching (FoSTeS) [12] or MM Break-Induced Replication (MMBIR) [13]. Either MMBIR or FoSTes could be the underlying mechanism for this mutational event.

Besides disrupting the coding sequences of a coincident gene, inversion breakpoints may alter the expression of adjacent genes by displacing their regulatory elements. The second breakpoint of the inversion detected in our patient was located in a large intergenic region, ~223 kb downstream of the *PTCHD1* gene and ~48 kb upstream of the *PRDX4* gene. Pathogenic variants in *PTCHD1* (MIM*300828) have been associated with susceptibility to autism (MIM #300830). Nonetheless, the autistic features of our patient could be primarily related to the dystrophinopathy itself, as there is known to be a higher incidence of neurodevelopmental disorders, such as autism spectrum and attention-deficit hyperactivity disorders, in D/BMD patients [14]. No disease-causing variants have been described in the *PRDX4* gene. Studies indicate that *PRDX4* might assist to prevent the progression of a metabolic syndrome by reducing local and systemic oxidative stress and synergistically suppressing steatosis, inflammatory reactions, and/or apoptotic activity [15]. Expression of both *PTCHD1* and *PRDX4* genes was tested and, although not quantified, there appeared to be no alteration in the patient as compared to a control sample (results not shown).

Intragenic inversions in the *DMD* gene have been described in seven patients, four of which were identified by array-CGH [16,17,18,19], two were detected by analysis of *DMD* transcripts [20,21] and one by long-read *DMD* sequencing [22]. The majority of cases corresponded to an inversion of one or two exons flanked by intronic deletions, resulting in exon skipping or pseudoexon inclusion in the mature mRNA. Regarding extragenic intrachromosomal inversions involving the *DMD* gene, four patients were previously described. Two of these presented a *DMD* phenotype associated with other syndromic features. They had complex chromosomal rearrangements involving not only an inversion disrupting the *DMD* gene, but also other inversions, gene duplications or contiguous gene deletions, which were detected by routine cytogenetic methods such as karyotyping and fluorescence in situ hybridization (FISH) [23,24]. In the other two patients, the inversion resulted in novel gene fusions. Wheway and collaborators (2003) described a BMD patient with a complex gene deletion syndrome. At one of the breakpoints, they observed a 600 kb inversion of *DMD* exons 53–79 combined with a 35 kb deletion removing *DMD* exon 52. At the other breakpoint, they found a ~1.6 Mb deletion, which removed four genes (*GKD*, *AHC*, *MAGEB* and *FTHL17*) and part of the mental retardation gene *IL1RAPL1* [25]. This deletion-inversion-deletion resulted in a chimeric *IL1RAPL1*-dystrophin transcript capable of translating into a hybrid protein. Tran et al. (2013) characterized a very large inversion in a *DMD* patient with mental retardation: inv(X)(p.21.2;q28). This inversion disrupted the *DMD* gene at intron 18, and the remaining dislocated *DMD* sequence originated a fusion transcript that enabled the identification of a novel non-coding gene—*KUCG1*—at the breakpoint on Xq28. The authors proposed that since *KUCG1* is expressed in the brain, its disruption may be responsible for the intellectual disability in the index case [26]. This case presents more similarities with our patient since it is the only inversion where no significant loss of genetic material was observed.

The present novel complex rearrangement involving *DMD* could not be detected by conventional diagnostic methods or by next-generation sequencing applications, such as gene panel or exome sequencing analysis. It is thus plausible that the contribution of such structural rearrangements as the underlying genetic cause in many diseases may be underestimated, considering that exome sequencing fails to detect truncated genes with deep intronic breakpoints. A comprehensive study, resorting to transcriptome analysis combined with WGS, was critical to reaching a final diagnosis for this BMD patient. Due to some experimental constraints, the average coverage data of WGS was ~9x (>30x is advisable for reliable single nucleotide polymorphism (SNP) and INDEL variant calling), and the average DNA molecule length was ~15 kb (>40 kb is now recommended for SV detection). The latter parameter probably explains why automated SV calling failed to detect the rearrangement involving the *DMD* gene.

In summary, we describe a novel disruptive structural rearrangement causing Becker Muscular Dystrophy. Besides expanding the *DMD* mutational spectrum, this work demonstrates the importance of introducing WGS into the clinical genetic diagnostic workup. Although an improvement of bioinformatic tools and comprehensive variant databases are still required to address the analytic and interpretative burden for the diagnostic laboratory, WGS has the unique advantage of detecting a wide variety of mutation types and the potential to become a “one-size-fits-all” genetic testing approach in the near future.

## 4. Materials and Methods

### 4.1. Case Report

The patient is a 23-year-old male presenting developmental delay, elevated creatine kinase levels, mild intellectual disability (with poor language skills and reduced social interaction), limb-girdle and scapular muscle atrophy (especially deltoids) and absence of Gowers’ sign. To date, no cardiac or respiratory involvement has been observed.

He was first referred to pediatric consultation at 2 years of age due to delayed motor milestones. At that time a muscle biopsy was performed, showing atrophy and hypertrophy of some fibers, discrete fibrosis and rare necrotic fibers. IHC studies were performed for dystrophin (DYS1, DYS2 and DYS3 fractions), alpha-, beta-, gamma- and delta-sarcoglycans, and merosin. Expression was absent for DYS2, irregular and reduced for DYS3 and diffusely reduced for DYS1 (Supplementary Material SI). The staining profile was normal with all remaining antibodies. A second deltoid muscle biopsy was performed at the age of twelve years, where IHC studies confirmed the altered dystrophin staining profile and revealed normal labeling for alpha-dystroglycan. Electromyography results were also compatible with myopathy. *DMD* gene analysis resorting initially to Southern-blot analysis and PCR multiplexed for 18 exons, showed no exonic deletions. Additional genetic studies were performed over the years, including karyotyping, array-CGH, subtelomeric MLPA screening, *FMR1* and *AFF2* molecular screening, *FKRP*, *SGCB*, *SGCG* and *SGCD* gene sequencing, all of which with normal results, the exception being a heterozygous variant of unknown clinical significance (VUS) detected in the *SGCA* gene. Data related to the case (variant and phenotype) have been submitted to the *DMD* gene variant database (https://databases.lovd.nl/shared/individuals/0000626404).

### 4.2. gDNA Sequencing

All 79 *DMD* exons and flanking intronic regions were amplified by PCR using M13-tailed primers. Amplicons were purified with Illustra ExoProStar 1-Step Kit (GE Healthcare, Buckinghamshire, UK) and sequenced using M13 universal primers and Big Dye Terminator v3.1 Cycle Sequencing Kit (Thermo Fisher Scientific, Waltham, MA, USA). Products were resolved on an ABI 3130xl Genetic Analyzer (Applied Biosystems, Foster City, CA, USA) and variant analysis was aided by Seqscape V2.5 (Thermo Fisher Scientific). Reference sequence for variants description: NM_004006.2.

### 4.3. Multiplex Western Blot

The multiplex Western blotting (WB) technique was modified from the protocol described by Anderson and Davison [27]. Cryopreserved muscle specimens were tested for abundance of dystrophin, dysferlin and four sarcoglycans (alpha, beta, gamma and delta) by semi-quantitative analysis, using immunoblotting and densitometry. Amounts were assessed as normal, reduced or absent in comparison to normal controls. Monoclonal antibodies (Novocastra, Leica Biosystems, Buffalo Grove, IL, USA) were used to detect the dystrophin rod (NCL-DYS1), C-terminal (NCL-DYS2) and N-terminal (NCL-DYS3) domains, as well as to detect dysferlin (NCL-Hamlet), the sarcoglycans alpha (NCL-a-SARC), beta (NCL-b-SARC), gamma (NCL-g-SARC) and delta (NCL-d-SARC), and beta-dystroglycan (NCL-b-DG), the latter used as an internal loading control. Primary antibodies were detected by ECL chemiluminescence (Thermo Fisher Scientific). Protein loading was normalized with the myosin band in the post-blotted gel.

### 4.4. Deletion/Duplication Screening (gDNA and cDNA)

Screening for deletions and duplications in the *DMD* gene was carried out by MLPA using two sets of probe mixes (P034 and P035, MRC-Holland, Amsterdam, the Netherlands) covering all exons as well as the Dp427 cortical promoter. MLPA analysis was performed in the patient and normal controls, initially on gDNA and later on muscle cDNA samples. A total of 100–150 ng of each sample were used and the protocol followed the manufacturer’s instructions. PCR products were run on an ABI 3130xl Genetic Analyzer (Applied Biosystems, Foster City, CA, USA). For data analysis, the GeneMarker Software (SoftGenetics, LLC, State College, PA, USA) was used, and the population normalization method and probe ratio plot were selected.

### 4.5. cDNA Sequencing

Total RNA was obtained from a cryopreserved muscle biopsy sample using PerfectPure RNA Fibrous Tissue kit (5 PRIME, Hamburg, Germany), and subsequently converted to cDNA using High Capacity cDNA Reverse Transcription kit (Thermo Fisher Scientific). *DMD* cDNA was amplified in 10 overlapping fragments and the resulting amplicons were sized on an agarose gel, purified using Illustra ExoProStar 1-Step kit (GE Healthcare, Buckinghamshire, UK) and sequenced with BigDye Terminator v3.1 Cycle Sequencing kit (Thermo Fisher Scientific). PCR products were purified using Dye-Ex 96 kit (Qiagen Inc., Valencia, CA, USA) and resolved on an ABI 3130xl Genetic analyzer (Applied Biosystems).

### 4.6. Low-Coverage Whole-Genome Sequencing (WGS)

The linked read library was prepared using the Chromium Genome Reagent Kit (10x Genomics) according to the manufacturer’s protocols (described in the Chromium Genome User Guide Rev A). Briefly, a total of 1.25 ng of gDNA was partitioned using the microfluidic Genome Chip to combine a library of Genome Gel Beads with template gDNA, in a mastermix using partitioning oil to create Gel Beads-in-Emulsion (GEMs). The droplets were isothermally incubated (3 h at 30 °C, 10 min at 65 °C and held at 4 °C) and barcoded fragments ranging from a few to several hundred base pairs were generated. After incubation, the GEMs were dissociated and the barcoded DNA fragments were recovered using Silane beads, and subsequently, size-selected using Solid Phase Reversible Immobilization (SPRI) beads for library preparation. Standard library preparation was according to the manufacturer’s instructions (described in the Chromium Genome User Guide Rev A) to construct sample-indexed libraries using 10x Genomics adaptors and to add the p5 and p7 sequences for Illumina sequencing. After quantification using qPCR (KAPA Biosystems Library Quantification Kit for Illumina platforms), the library was sequenced using an Illumina HiSeq 4000 with 2 × 150 paired-end reads based on the manufacturer’s protocols. The 10x Long Ranger software v2.1.6 (10x Genomics, Pleasanton, CA, USA) was used for SV automated calling and 10x Loupe browser was used for visualization. For a more detailed NGS data analysis, so as to identify the SV breakpoints, the BAM file was visually inspected using IGV from Broad Institute [7].

### 4.7. Breakpoint Characterization

Specific primers were designed to confirm the inversion breakpoints and perform SV screening (BreakPoint1-F: ATGGGCATCAGTCAGGCTTTC; BreakPoint1-R: CCCTGTGTTCTGGCTATGCTG; BreakPoint2-F: TACAGTTTCCAAAGGGCAAGAC; BreakPoint2-R: CAGTCCATGCCCCAAAGAC). Amplified products were purified and sequenced as described above.

### 4.8. Transcript Analysis

To further characterize the aberrant *DMD* transcripts, several specific primers were designed to target the *DMD* exon/intron 74 and the *PRDX4* upstream regions (primer sequences available on request). PCR assays were set up using different primer combinations. Amplicons were resolved on an agarose gel and sequenced as described above.

### 4.9. PRDX4 and PTCHD1 Expression

Expression of *PRDX4* (peroxidase cytoplasmic protein) and *PTCHD1* (Patched domain-containing protein 1) was evaluated using cDNA samples obtained from muscle biopsies of the patient and a normal control. Primers were designed to target exons 1 to 4 of *PRDX4* (cPRDX4-1-4-F: TGCCGCTACTGCTGTTCCTG; cPRDX4-1-4-R: TCCTTATTGGCCCAAGTCCTC) and exons 2 and 3 of *PTCHD1* (cPTCHD1-2-3-F: AGCCGCGTATCAGAACGTTAC; cPTCHD1-2-3-R: AATGTTCGTGAAAGGGCTGG). Amplified products were visualized on an agarose gel and sequenced as described.

### 4.10. Rearrangement Screening

Structural variant segregation analysis was performed by screening the patient’s mother (the only available family member). One PCR assay targeted the inversion breakpoint sequence and the other targeted the intron 74 reference sequence in the same region (wildtype allele). Primer pairs were those used for breakpoint characterization (above) and the following pair for the wildtype sequence: int74_WT-F–TTGCCAGAACCAAGACCCATC; int74_WT-R–AGATACAGTCCATGCCCCAAAG. A total of 80 uncharacterized B/DMD patients were also screened for the presence of the inversion breakpoint sequence.

## Data Availability

Data supporting the findings of this study not presented within the article or its supplementary materials, are available upon request.

## Supplementary Materials

The supplementary materials are available online at https://www.mdpi.com/article/10.3390/ijms23010059/s1.
