# Supplementary material for: Integrating Whole-Genome Sequencing in Clinical Genetics: A Novel Disruptive Structural Rearrangement Identified in the Dystrophin Gene (DMD)
Source: Int J Mol Sci. 2021 Dec 22;23(1):59. doi: 10.3390/ijms23010059 (PMC8744749; doi:10.3390/ijms23010059)
Supplement: Supplementary file 1 [file ijms-23-00059-s001.zip › Supporting Information files_Original Images_revision.pdf]

Supporting Information files

a) Original Images of Western Blot - Supplementary Material III

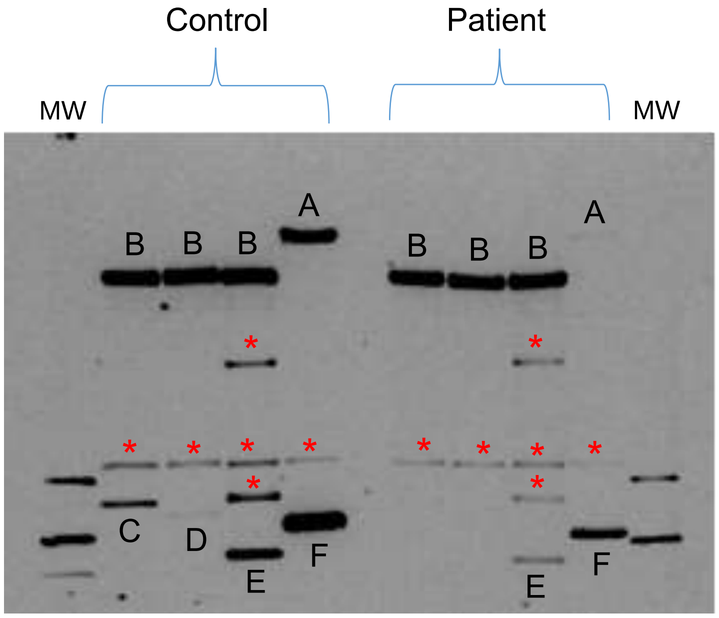

A – dystrophin (DYS1)
B – dysferlin
C – alfa-sarcoglycan
D – beta-sarcoglycan
E – gamma-sarcoglycan
F – beta-dystroglycan
* - unspecific bands
MW - protein standards

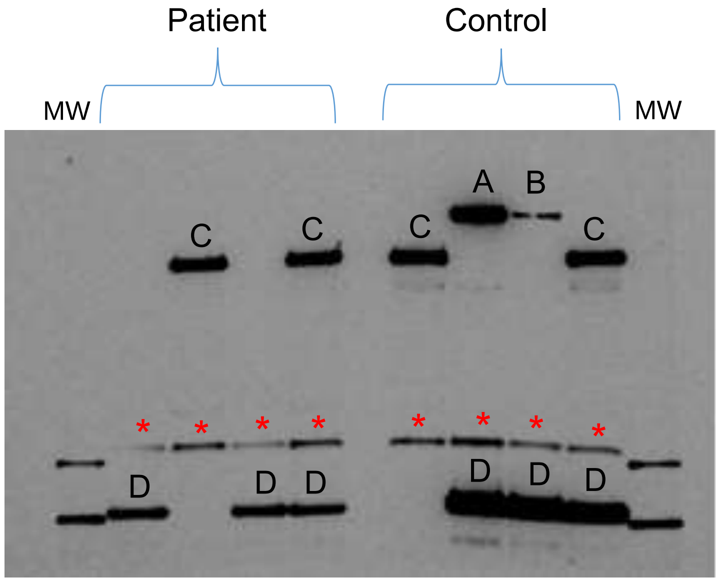

A – dystrophin (DYS2)
B – dystrophin (DYS3)
C – dysferlin
D – beta-dystroglycan
* - inespecific bands
MW - protein standards

b) Original Image of gel - Supplementary Material IV

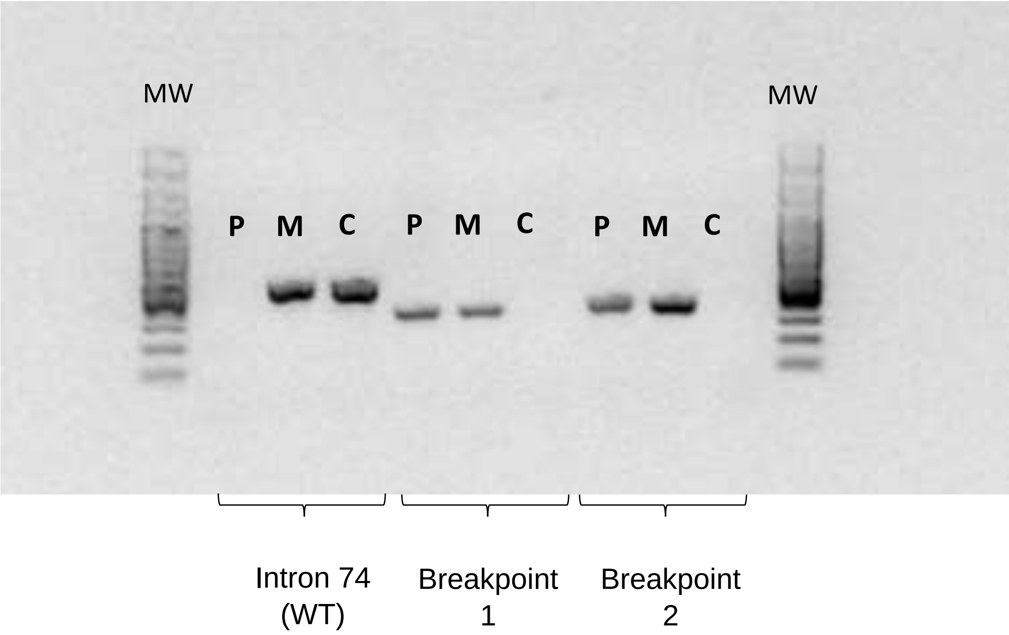

WT – Intron 74 reference sequence
P – Patient
M – Mother
C – Male control DNA
MW – molecular weight marker
