# Supplementary material for: Integrating Whole-Genome Sequencing in Clinical Genetics: A Novel Disruptive Structural Rearrangement Identified in the Dystrophin Gene (DMD)
Source: Int J Mol Sci. 2021 Dec 22;23(1):59. doi: 10.3390/ijms23010059 (PMC8744749; doi:10.3390/ijms23010059)
Supplement: Supplementary file 1 [file ijms-23-00059-s001.zip › Supplementary Materials.zip!/Supplementary Material I.pdf]

Supplementary Material I

A 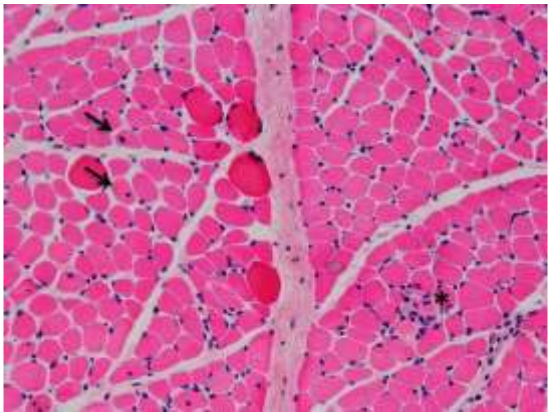

B 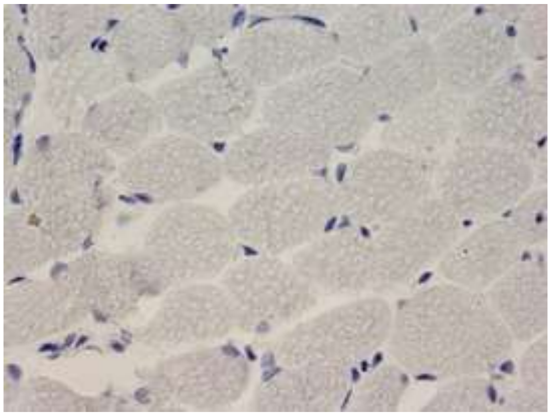

Patient`s muscle biopsy. A) The histological examination of slides stained with hematoxylin and eosin (H&E, 100x) revealed a slight to moderate change in muscle architecture. It was observed a diffuse variation in the size of muscle fibers, with hypertrophic and atrophic fibers with round and regular contours, and a slight increase in connective tissue. A moderate increase in internal nuclei (arrows) and occasional necrotic fibers were also identified (*). B) The immunohistochemical study revealed complete loss of expression of the DYS2 fraction of dystrophin (400x).
