# Supplementary material for: Integrating Whole-Genome Sequencing in Clinical Genetics: A Novel Disruptive Structural Rearrangement Identified in the Dystrophin Gene (DMD)
Source: Int J Mol Sci. 2021 Dec 22;23(1):59. doi: 10.3390/ijms23010059 (PMC8744749; doi:10.3390/ijms23010059)
Supplement: Supplementary file 1 [file ijms-23-00059-s001.zip › Supplementary Materials.zip!/Supplementary Material II.pdf]

Supplementary Material II

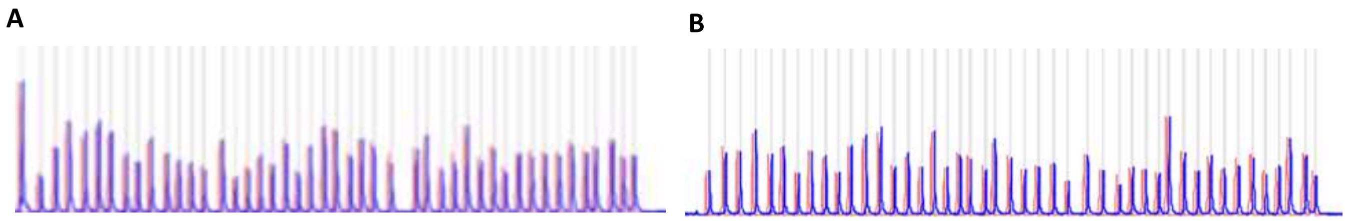

Patient`s MLPA results (gDNA). No deletions or duplications were detected in the *DMD* gene using both probes from MRC-Holland (A-P034; B-035). Patient fragments are shown in blue and control fragments in red.

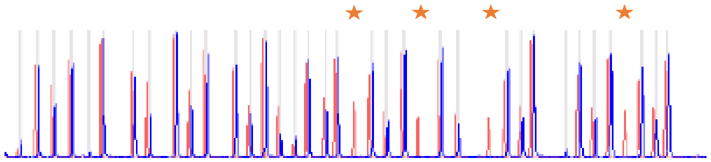

Patient`s MLPA results (cDNA). MLPA probe 034 showed absence of signal from probes detecting the sequence corresponding to exons 75, 76, 77 and 79 (orange stars) of *DMD* gene.
