# Supplementary material for: Integrating Whole-Genome Sequencing in Clinical Genetics: A Novel Disruptive Structural Rearrangement Identified in the Dystrophin Gene (DMD)
Source: Int J Mol Sci. 2021 Dec 22;23(1):59. doi: 10.3390/ijms23010059 (PMC8744749; doi:10.3390/ijms23010059)
Supplement: Supplementary file 1 [file ijms-23-00059-s001.zip › Supplementary Materials.zip!/Supplementary Material III.pdf]

Supplementary Material III

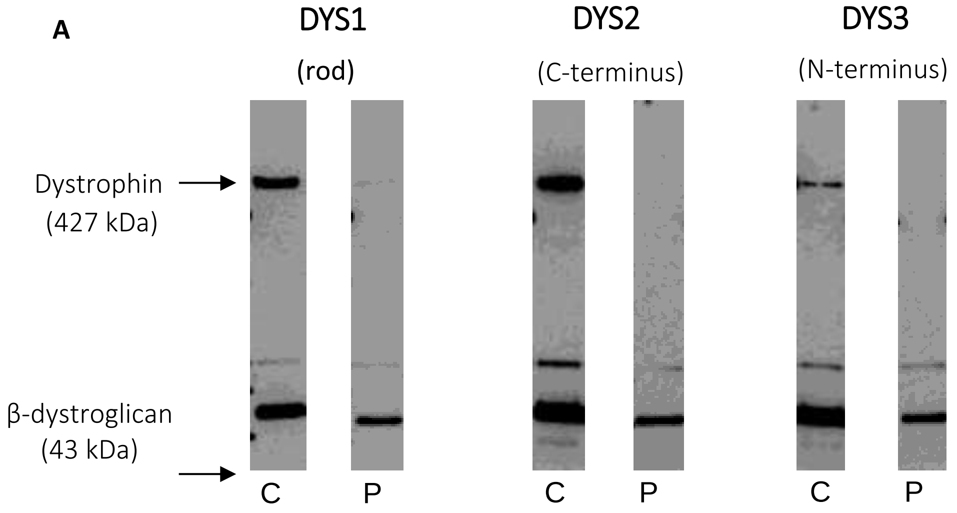

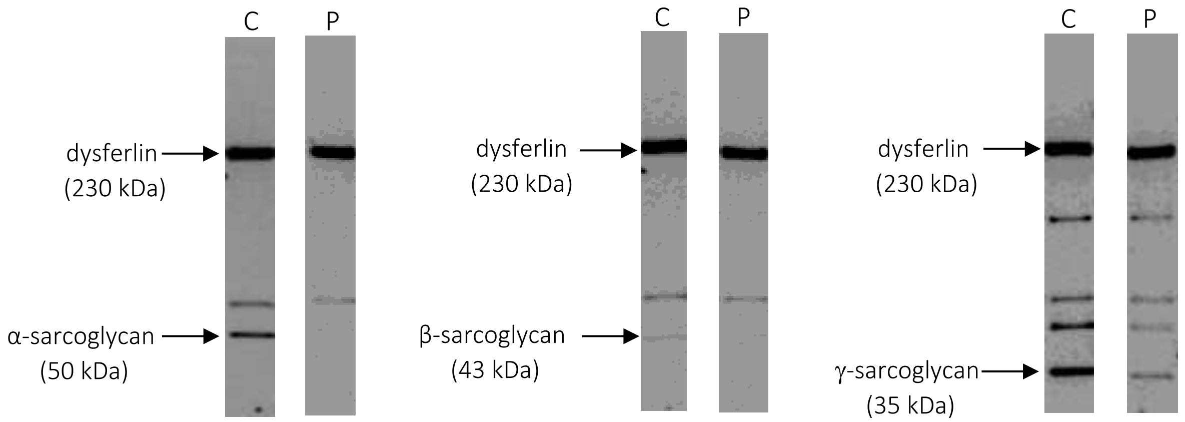

Western Blot results. A) Absence of labelling for two of the dystrophin antibodies (DYS2 and DYS3) and reduction for DYS1. B) α-sarcoglycan and β -sarcoglycan also undetectable (C-Control; P-Patient).
