# Supplementary material for: Integrating Whole-Genome Sequencing in Clinical Genetics: A Novel Disruptive Structural Rearrangement Identified in the Dystrophin Gene (DMD)
Source: Int J Mol Sci. 2021 Dec 22;23(1):59. doi: 10.3390/ijms23010059 (PMC8744749; doi:10.3390/ijms23010059)
Supplement: Supplementary file 1 [file ijms-23-00059-s001.zip › Supplementary Materials.zip!/Supplementary Material IV.pdf]

Supplementary Material IV

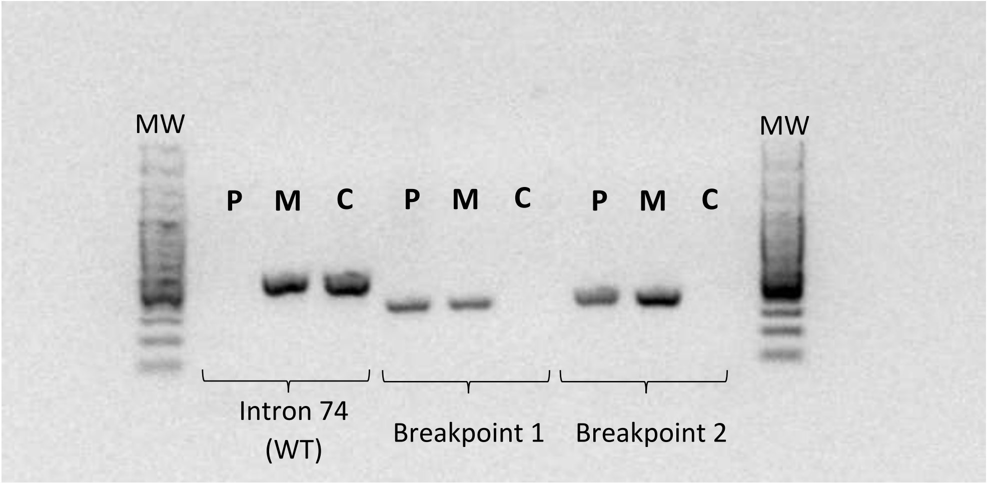

Family segregation studies. PCR amplification of intron 74 reference sequence (WT) and both inversion breakpoints in patient (P), his mother (M) and a male control DNA (C), shows the presence of the inversion in the mother in a heterozygous state (MW- molecular weight marker).
