# Supplementary material for: Integrating Whole-Genome Sequencing in Clinical Genetics: A Novel Disruptive Structural Rearrangement Identified in the Dystrophin Gene (DMD)
Source: Int J Mol Sci. 2021 Dec 22;23(1):59. doi: 10.3390/ijms23010059 (PMC8744749; doi:10.3390/ijms23010059)
Supplement: Supplementary file 1 [file ijms-23-00059-s001.zip › Supplementary Materials.zip!/Supplementary Material V.pdf]

Supplementary Material V

PRDX4_WT	GAGTTTCTGCCAGCATTAGCCCATTAAGCCATGTCAGAAATATAAAATAGTTGAGGTAGAAGAGA
DMD_WT	CTCCATCACACTGCTCAAAATAACTTTCCCCATGGAGCCCTGCTAGATCCCTGCAGTTGGAAGAAATCTGTCTTT
BrPT-1	GAGTTTCTGCCAGCATTAGCCCATTAGCCCTGCTAGATCCCTGCAGTTGGAAGAAATCTGTCTTT
BrPT-2	CTCCATCACACTGCTCAAAATAACTTACATTAGCCCATGTCAGAAATTTCTAACATTAGCCCATGTCAGAAATAAAATAGTTG..

Alignment of sequences at inversion junctions. Microhomology was found between the wild type sequences in the breakpoint regions (boxed). This microhomology is absent in one of the breakpoints (BrPT-1), and part of a duplicated region (underlined) in the other breakpoint (BrPT-2).
